# Late Diagnosis of Infants with PCD and Neonatal Respiratory Distress

**DOI:** 10.3390/jcm9092871

**Published:** 2020-09-04

**Authors:** Myrofora Goutaki, Florian S. Halbeisen, Angelo Barbato, Suzanne Crowley, Amanda Harris, Robert A. Hirst, Bülent Karadag, Vendula Martinu, Lucy Morgan, Christopher O’Callaghan, Ugur Ozçelik, Sergio Scigliano, Santiago Ucros, Panayiotis Yiallouros, Sven M. Schulzke, Claudia E. Kuehni

**Affiliations:** 1 Institute of Social and Preventive Medicine, University of Bern, 3012 Bern, Switzerland; myrofora.goutaki@ispm.unibe.ch (M.G.); florian.halbeisen@ispm.unibe.ch (F.S.H.); 2 Paediatric Respiratory Medicine, Children’s University Hospital of Bern, University of Bern, 3010 Bern, Switzerland; 3 Primary Ciliary Dyskinesia Centre, Department of Women’s and Children’s Health (SDB), University of Padova, 1848 Padova, Italy; angelo.barbato45@gmail.com; 4 Paediatric Department of Allergy and Lung Diseases, Oslo University Hospital, 0372 Oslo, Norway; suzcro@ous-hf.no; 5 Primary Ciliary Dyskinesia Centre, NIHR Respiratory Biomedical Research Centre, University of Southampton, Southampton SO 16 6YD, UK; Amanda-Lea.Harris@uhs.nhs.uk; 6 Department of Respiratory Sciences, College of Life Sciences, University of Leicester, Leicester LE1 7RH, UK; rah9@leicester.ac.uk (R.A.H.); c.ocallaghan@ucl.ac.uk (C.O.); 7 Department of Pediatric Pulmonology, Marmara University, School of Medicine, Istanbul 34854, Turkey; bkaradag@hotmail.com; 8 Department of Paediatrics, 2nd Faculty of Medicine, Charles University and University Hospital Motol, 38FQ+FX Prague, Czech Republic; vendy.martinu@gmail.com; 9 Department of Respiratory Medicine, Concord Hospital Clinical School, University of Sydney, Sydney, NSW 2138, Australia; Lucy.Morgan@health.nsw.gov.au; 10 Respiratory Critical Care & Anaesthesia, UCL Great Ormond Street Institute of Child Health, London WC1N 1EH, UK; 11 Department of Pediatric Pulmonology, Hacettepe University Faculty of Medicine, 06230 Ankara, Turkey; uozcelik@hacettepe.edu.tr; 12 Centro Respiratorio, Hospital de Niños Ricardo Gutierrez, Buenos Aires C1425EFD, Argentina; sergioscigliano@gmail.com; 13 Departamento de Pediatría, Fundación Santa Fe, Clínica de Neumología Pediátrica Compensar, Bogotá 12390, Colombia; santiago_ucros@yahoo.com; 14 Medical School, University of Cyprus, 596G+R2 Nicosia, Cyprus; yiallouros.panayiotis@ucy.ac.cy; 15 Department of Neonatology, University Children’s Hospital Basel UKBB, 4056 Basel, Switzerland; sven.schulzke@unibas.ch

**Keywords:** primary ciliary dyskinesia, neonatal respiratory distress, laterality defect, orphan diseases

## Abstract

Neonatal respiratory distress (NRD) is common among infants with primary ciliary dyskinesia (PCD), but we do not know whether affected neonates receive a timely diagnosis. We used data from the international PCD cohort and assessed the proportion of patients with PCD who had a history of NRD and their age at diagnosis, stratifying by presence of laterality defects. First we analyzed data from all participants diagnosed after 2000, followed by individuals from a subgroup diagnosed using stricter criteria. Among the 1375 patients in the study, 45% had a history of NRD and 42% had laterality defects. Out of the 476 children with definite PCD diagnosis, 55% had a history of NRD and 50% had laterality defects. Overall, 30% of children with PCD were diagnosed during the first 12 months of life. This varied from 13% in those with situs solitus and no NRD, to 21% in those with situs solitus and NRD, 33% in those with situs anomalies but no NRD, and 52% in those with both situs anomalies and NRD. Our results suggest that we need to improve our knowledge of the neonatal presentation of infants with PCD and apply it so that these patients will receive appropriate care sooner.

## 1. Introduction

Primary ciliary dyskinesia (PCD) is a multiorgan genetic disease that affects approximately 1 in 10,000 people [1,2], or as many as 1 in 400 in highly consanguineous populations [3,4]. The clinical phenotype is variable, but most patients have chronic upper and lower airway disease with rhinitis and cough resulting in recurrent infections of ears, sinuses, and lungs [5,6]. About half of patients with PCD have situs inversus, and an additional 10–12% have other laterality defects, which may be associated with congenital heart disease [7,8]. Already in childhood, lung function is comparable to patients with cystic fibrosis (CF), and as the disease progresses most patients develop progressive lung disease with bronchiectasis and chronic pseudomonas infection [9,10,11,12,13]. At advanced disease stages, many adults become oxygen dependent and undergo a lobectomy, with some eventually requiring lung transplantation [14,15]. It is believed that, as in other genetic respiratory diseases such as CF, early diagnosis followed by initiation of regular physiotherapy and prompt antibiotic treatment may reduce lung function decline and improve long-term outcomes [16].

Patients with PCD are usually born at term, and typically present with chronic respiratory symptoms from birth such as rhinitis and a wet-sounding cough. A large proportion present with neonatal respiratory distress (NRD) [17,18,19]. The frequency of these symptoms in neonates with PCD is unclear; in a systematic review, the proportion of PCD patients reported to have NRD varied widely between studies, from 15% to 91%. However, these data were generally of poor quality [20]. In a single-center case-control study from Canada, detailed neonatal data were extracted from health records to identify characteristics that distinguish PCD from other causes of NRD [19]. The study showed that compared to other term infants with NRD, infants with PCD had a characteristic clinical picture that should point toward the diagnosis [19].

The neonatal period offers an ideal window for early diagnosis of PCD before long-term damage to the lungs has occurred. However, timely diagnosis has not yet been investigated in an international setting. We used a multicenter dataset from the international PCD (iPCD) cohort to determine the proportion of patients presenting with NRD and the age when PCD was finally diagnosed. We grouped infants into those with and without NRD, and stratified additionally by presence of laterality defects.

## 2. Methods

### 2.1. Participants

**Figure 1:**
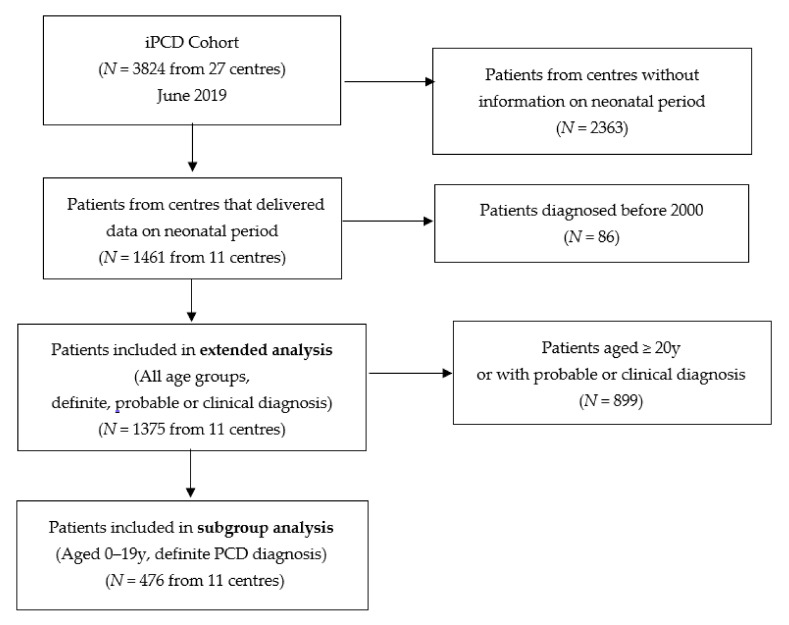
Flow chart showing the patients included for the different analyses performed. PCD: primary ciliary dyskinesia.

The iPCD cohort, described in detail elsewhere [21], included 3824 patients as of June 2019 when reviewed for this study [21]. The core dataset of the iPCD cohort is compulsory for all contributors, while specific modules including the one on neonatal history are voluntary. For this analysis, we included only patients from data providers who had also contributed data on the neonatal period for their patients (Figure 1 and Supplementary Materials Table S1). We further excluded patients diagnosed before the year 2000 because PCD diagnosis has evolved considerably during the past 20 years and because we were mainly interested in recent data that are representative of contemporary neonatal care [22].

### 2.2. Definition of NRD and PCD Diagnosis

Information on NRD was delivered by data providers and was retrieved from patient charts. NRD was described as a history of hospitalization for respiratory distress during the neonatal period either retrieved from neonatal hospital records or reported to the PCD clinic at diagnosis by patients or their parents. In one of the centers, NRD was defined as the presence of respiratory distress or physician-recorded chest symptoms during the neonatal period, since the data did not distinguish between the two. We also retrieved information on laterality status and defined the presence of situs inversus or heterotaxia as a laterality defect. All patients included in the study had a clinical phenotype consistent with PCD and were under PCD management at the contributing centers.

All patients included in the iPCD cohort had a strong clinical suspicion of PCD and were followed up as PCD cases in the contributing centers after alternative diagnoses such as CF or immunodeficiency had been excluded. However, because the diagnosis of PCD is complex and can sometimes remain inconclusive even after multiple tests, we grouped patients into groups of diagnostic certainty based on the diagnostic guidelines of the European Respiratory Society Task Force [23].

“Definite PCD” diagnosis was defined as a pathogenic biallelic PCD genetic mutation or hallmark structural abnormality in transmission electron microscopy (TEM) [23]. The remaining patients had either abnormal high-speed video microscopy findings or low nasal nitric oxide (probable PCD diagnosis), or were patients with strong clinical suspicion (e.g., Kartagener syndrome) in whom the diagnostic algorithm was not complete (clinical PCD diagnosis). For the diagnostic classification, we reviewed all diagnostic data provided by the centers and contacted the centers where clarifications were required. Age at diagnosis was defined based on information provided by the centers or, when not available, based on the date of positive diagnostic tests. In case several positive tests were available, age of diagnosis corresponded to the date of the first positive test. For patients with only clinical diagnosis, age of diagnosis was defined as the age when the patient started to be managed as PCD at the participating center. We divided the ages at diagnosis into periods, to investigate whether the diagnosis was made in the first three months of life, within infancy (age 3–11 months), during the preschool period (age 1–4 years), or during school years (age 5–9, 10–14, or over 14 years).

### 2.3. Analysis

We first analyzed data from all patients regardless of age or diagnostic certainty (Figure 1), and assessed the proportions of patients who reported NRD and laterality defects overall and by country. We then stratified the population into four groups and described their ages at diagnosis: patients without NRD and with situs solitus, patients with NRD and situs solitus, patients without NRD and with laterality defects, and patients with both NRD and laterality defects. We assessed the proportion of children diagnosed with PCD within the first three and the first 12 months of life, and the proportion diagnosed later at ages 1–4, 5–9, 10–14, or 15–19 years. We also compared age at diagnosis using a Kruskal–Wallis test [24], followed by pairwise comparisons between groups using a Wilcoxon rank sum test [25].

Second, we repeated all analyses in patients who had “definite PCD” based on recent diagnostic guidelines and were younger than 20 years at the time of investigation, because recall of neonatal problems declines with age (subgroupanalysis, Figure 1) [23,26]. We used STATA 15.1 (StataCorp, College Station, TX, USA) for all analyses. While the first dataset is representative of the majority of patients with PCD currently in treatment, the second includes those for whom quality of information on PCD diagnosis and the neonatal period is best.

### 2.4. Ethics

In most participating countries, researchers are not required to obtain patient informed consent for retrospectively collected anonymized observational data. In countries where informed consent was needed, primary investigators obtained local ethics approval and informed consent for the contribution of their anonymized data to the iPCD cohort for research purposes [21]. In Switzerland, contribution of anonymized data were approved by the Cantonal Ethics Committee of Bern (KEK-BE: 060/2015).

## 3. Results

Out of the 3824 patients included in the iPCD cohort at the time of the study, 1461 originated from centers that completed also the module on the neonatal period (Figure 1). After excluding patients diagnosed earlier than 2000, 1375 patients were included in the study (extended analysis). The subgroup analysis included 476 patients who were 0–19 years old and had a definite PCD diagnosis (Figure 1). Data came from 11 centers in nine countries: Argentina, Australia, Colombia, Cyprus, Czech Republic, Italy, Norway, Turkey, and the United Kingdom (UK). Seven countries were represented by one center, while Turkey and UK each had two contributing centers.

Of the entire study population (*n* = 1375), 45% (95% Confidence Interval (CI): 43–48%) had a history of NRD and 42% (95% CI: 40–45%) a laterality defect (Supplementary Materials Table S2). PCD was diagnosed at a median age of 9.8 years. Only 13% of the 1375 were diagnosed during the first 12 months of life, varying from 4% in those with situs solitus and no NRD to 32% in those with NRD and a laterality defect (Table S2). Median age at diagnosis was 12.4 years for patients without NRD and situs solitus, 10.4 years for those with NRD and situs solitus, 8.8 years for those without NRD but with a laterality defect, and 4.5 years for the group combining a history of NRD and laterality defects. In 14% of patients with NRD and a laterality defect, PCD was diagnosed in the first three months of life (Supplementary Materials Table S3). Among patients with NRD but without a laterality defect, PCD was only diagnosed in the first three months of life only in 2.9%. We found a difference in median age at diagnosis between male and female patients (2.6 compared to 4.4 years) but it disappeared after accounting for NRD and laterality defects.

Results were comparable in the subgroup analyses that included only patients 0–19 years with definite PCD diagnosis. Fifty-five percent (95% CI: 50–59%) reported a history of NRD and 50% (95% CI: 46–55%) had a laterality defect (Table 1). Prevalence of NRD and laterality defects varied between centers (Supplementary Materials Table S4). Age at diagnosis overall was lower than in the whole study population and the differences between the four groups more pronounced. Patients were diagnosed at a median age of 3.4 years, varying from less than one year in Norway and Cyprus to 10 years in Turkey (Table 1). Overall, 30% of children with PCD were diagnosed during the first 12 months of life, varying from 13% in those with no NRD and situs solitus, to 21% in those with NRD and situs solitus, 33% in those with a laterality defect but no NRD, and 52% in those with both NRD and a laterality defect.

**Figure 2:**
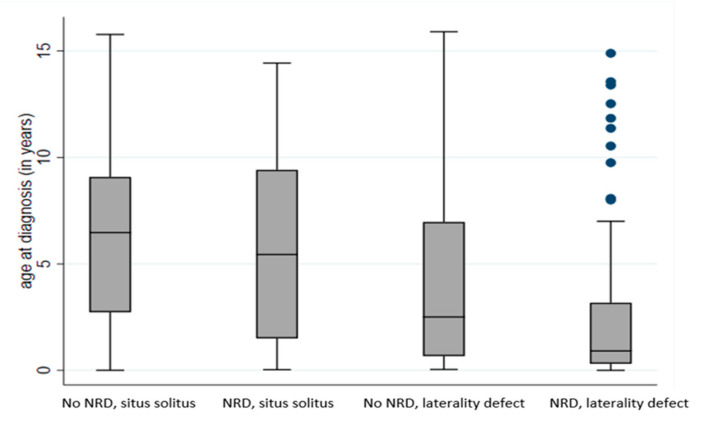
Age at diagnosis of PCD patients (*n* = 476) aged 0–19 years with definite PCD diagnosis, per clinical characteristics group. PCD: primary ciliary dyskinesia, NRD: neonatal respiratory distress.

Table 2 and Figure 2 show a detailed breakdown of age at diagnosis for the four patient groups. For infants with NRD and a laterality defect, median age at diagnosis was 0.9 years (11 months): 21% were diagnosed within three months of birth, a further 31% before the age of 12 months, and most (80%) before they reached five years. Children with laterality defects not presenting with neonatal respiratory symptoms were also diagnosed relatively early (median age 2.5 years): one-third were diagnosed during infancy and 57% before the age of five years. These results stand in contrast with those for infants with situs solitus. Among infants who presented with NRD and situs solitus, only 6% were diagnosed with PCD within three months, 20% within the first year of life, and only 39% by age five. Of the children who had situs solitus, PCD was diagnosed at a median age of 5.4 years if they had NRD, but of 6.5 years if they had no neonatal symptoms (*p* = 0.15, Figure 2), suggesting that neonatal symptoms alone, in the absence of situs anomalies, do not lead to diagnostic testing for PCD.

Each box represents the median and interquartile range (IQR) of age at diagnosis for the respective group (in years). The whiskers represent the range of age at diagnosis and the dots represent outliers; pairwise comparisons between the four groups using a Wilcoxon rank sum test corrected for multiple testing (Benjamini–Hochberg) resulted in *p* ≤ 0.003, with the exemption of the comparison between infants with situs solitus and no NRD and infants with situs solitus and NRD (*p* = 0.150).

## 4. Discussion

This is the first large multinational study that describes the proportion of infants with PCD who presented with respiratory distress after birth and the age they were diagnosed. Among the 55% of children with PCD in the iPCD cohort who reported NRD, those who also had situs inversus were diagnosed with PCD relatively early, at a median age of 0.9 years. Children who had no laterality defect were diagnosed at a median age of 6 years, independently of whether or not they had presented with NRD. This suggests that although NRD in infants with PCD has typical features, the diagnosis is usually missed by neonatologists and pediatricians unless patients also present with situs anomalies.

The iPCD cohort is the largest dataset on patients with PCD worldwide, and is representative of and follows currently diagnosed PCD patients in developed countries [21]. We analyzed the whole dataset, which was large and representative of the majority of PCD patients in medical follow-up; then, to reduce uncertainties in diagnosis of PCD and of recall bias regarding the neonatal presentation, we repeated all analyses for patients with a definite diagnosis of PCD based on current diagnostic criteria, including those who were younger than 20 when neonatal history was taken. Still, our study has limitations. In particular, since information on NRD was collected retrospectively, there was a lack of standardization for the definition of NRD between centers, and for some participants this information was patient- or parent-reported. This could possibly explain the lower prevalence of NRD compared to previous studies [17,18,19]. We also lacked detailed information on the characteristics of the neonatal disease presentation, such as onset and duration of respiratory distress, exact diagnoses and treatments given at the hospital, and results of x-rays and other investigations. Thus, we cannot state whether the clinical picture of NRD was as typical as that previously described [19]. There are few other studies to compare our findings with. Prevalence of NRD in our study was lower than in a single-center study from Canada (91%), which was based on a chart review of neonatal records [19]. Similarly, a multicenter study including 205 children with PCD from North American centers reported a higher (81%) prevalence of NRD [18]. In our study, neonatal history was usually obtained from the patients or the parents at the time of PCD diagnosis. NRD prevalence varied between countries and was low in some centers. In several centers the prevalence of NRD was higher (e.g., 68–100%), suggesting that the recorded average might be an underestimation due to a less detailed history in some centers. In another study of consecutively referred and diagnosed patients with PCD from the UK, 56 of 75 patients (75%) reported neonatal chest symptoms [17]. As in our study, these data were also collected from patients or their parents at the first diagnostic visit. The prevalence found was higher than ours (45%). A possible explanation for this difference might be that the definition used for NRD in the UK study was wider, as it included also other neonatal chest symptoms, while in our study it included only history of NRD (with the exception of one center).

What implication does this have for clinical management? Given that up to 5% of term-born infants present with some type of respiratory distress and assuming that 50% of infants with PCD present with NRD while estimating prevalence of PCD as 1 in 10000, it follows that around 1 in 500 term neonates with NRD has PCD [27]. Performing the complex and expensive set of diagnostic tests to diagnose one infant with PCD in 500 is unrealistic, if not impossible. However, the data from the Canadian case-control study suggests (though this needs to be confirmed in other populations) that the clinical picture of NRD is quite typical in PCD, and it differs substantially from much more common diagnoses such as transient tachypnea of the newborn or peripartum pneumothorax, given that infants with PCD often required supplemental oxygen for several days to weeks and had lobar collapse. In that study, the combination of oxygen therapy for more than two days and lobar collapse in the chest x-ray had a sensitivity of 83% for detecting PCD [19]. Thus, term-born infants with late onset but long-duration respiratory distress not needing intubation, and with a radiological picture typical for PCD, could be picked up as suspected PCD candidates and referred for diagnosis from the first days of life. In our study, the average age at diagnosis for children in some centers (e.g., in Norway and Cyprus was 7–8 months) suggests that, although the number of patients was low, earlier diagnosis is possible.

## 5. Conclusions

We found that only a minority of infants with PCD who present typical symptoms in the neonatal period are diagnosed early, but examples from some countries suggest early diagnosis is possible. In term neonates with NRD, better recognition of the typical clinical picture associated with PCD during the neonatal period could increase the proportion of neonates referred for detailed PCD diagnostic testing. We believe that if the typical clinical picture of PCD in neonates can be better characterized and its recognition improved during the neonatal period, more infants suffering from PCD could receive appropriate care sooner.

## Figures and Tables

**Table 1:** Characteristics of PCD patients aged 0–19 years with a definite PCD diagnosis (*n* = 476).

Characteristic	*N* (%)	Age at Diagnosis (in Years)	*N* (%) of Patients Diagnosed at 0–12 m
		Median (IQR)	
**Total**	476 (100)	3.36 (0.69–7.76)	144 (30.3)
**Sex**			
Male	262 (55.0)	2.62 (0.61–7.06)	87 (33.2)
Female	214 (45.0)	4.38 (0.97–8.34)	57 (26.6)
**Country of residence**			
Argentina	30 (6.3)	1.5 (0.50–5.00)	10 (33.3)
Australia	19 (4.0)	1.26 (0.40–3.78)	9 (47.4)
Colombia	11 (2.3)	6.61 (4.04–8.34)	1 (9.1)
Cyprus	5 (1.0)	0.61 (0.21–0.67)	4 (80.0)
Czech Republic	21 (4.4)	4.75 (1.49–9.28)	4 (19.0)
Italy	89 (18.7)	1.62 (0.38–5.67)	36 (40.4)
Norway	25 (5.3)	0.63 (0.10–7.17)	14 (56.0)
Turkey	31 (6.5)	10.11 (7.37–12.53)	0 (0.0)
United Kingdom	245 (51.5)	3.53 (0.89–8.08)	66 (26.9)
**NRD**			
No	193 (40.6)	4.96 (1.10–8.44)	43 (22.3)
Yes	261 (54.8)	1.96 (0.50–6.40)	98 (37.5)
No information	22 (4.6)	2.47 (1.42–9.40)	3 (13.6)
**Organ laterality**			
Situs solitus	218 (45.8)	5.93 (2.00–9.08)	37(17.0)
Laterality defect	240 (50.4)	1.44 (0.42–5.07)	103 (42.9)
No information	18 (3.8)	6.35 (1.09–9.54)	4 (22.2)
**Clinical characteristics groups**			
No NRD, situs solitus	114 (24.0)	6.47 (2.73–9.08)	15 (13.2)
NRD, situs solitus	132 (27.7)	5.44 (1.50–9.41)	27 (20.5)
No NRD, laterality defect	91 (19.1)	2.51 (0.67–6.97)	30 (33.0)
NRD, laterality defect	139 (29.2)	0.91 (0.31–3.17)	72 (52.0)

PCD: primary ciliary dyskinesia, NRD: neonatal respiratory distress, IQR: interquartile range.

**Table 2:** Age at diagnosis of PCD patients aged 0–19 years with definite PCD diagnosis, per clinical characteristics group, based on reported NRD and organ laterality (*n* = 476).

Clinical Characteristics Groups	Age at Diagnosis *n* (%)						
	0–2 m	3–11 m	1–4 yrs	5–9 yrs	10–14 yrs	> 14 yrs	Total
**No NRD, situs solitus**	2 (1.7)	13 (11.4)	24 (21.1)	46 (40.4)	26 (22.8)	3 (2.6)	114 (100)
**NRD, situs solitus**	8 (6.1)	19 (14.4)	25 (18.9)	45 (34.1)	34 (25.7)	1 (0.8)	132 (100)
**No NRD, laterality defect**	6 (6.6)	24 (26.3)	22 (24.2)	27 (29.7)	11 (12.1)	1 (1.1)	91 (100)
**NRD, laterality defect**	29 (20.9)	43 (30.9)	39 (28.1)	20 (14.4)	7 (5.0)	1 (0.7)	139 (100)
**TOTAL**	45 (9.4)	99 (20.8)	110 (23.1)	138 (29.0)	78 (16.4)	6 (1.3)	476 (100)

PCD: primary ciliary dyskinesia, NRD: neonatal respiratory distress, m: months, yrs: years. Age 0–2 months corresponds to 0–2.999 months, similar for all age groups.

## Supplementary Materials

The following are available online at https://www.mdpi.com/2077-0383/9/9/2871/s1, Table S1: Inclusion criteria for the total study population and for the subgroup of younger patients with definite PCD, Table S2. Characteristics of PCD patients diagnosed after 2000 (*n* = 1375), Table S3. Age at diagnosis of PCD patients of all ages, per clinical characteristics group, based on reported NRD and organ laterality (*n* = 1375), Table S4. Prevalence of NRD and laterality defects in PCD patients aged 0–19 years with definite PCD diagnosis by country (*n* = 476).
