# Supplementary material for: Late Diagnosis of Infants with PCD and Neonatal Respiratory Distress
Source: J Clin Med. 2020 Sep 4;9(9):2871. doi: 10.3390/jcm9092871 (PMC7563976; doi:10.3390/jcm9092871)
Supplement: Supplementary file 1 [file jcm-09-02871-s001.pdf]

Table S1. Inclusion criteria for the total study population and for the subgroup of younger patients with definite PCD.

**Total study population (*N* = 1375)**	• Patients from centres that delivered data on neonatal period<br>• Diagnosed in 2000-2019<br>• All age groups<br>• Definite, probable or clinical PCD diagnosis
**Subgroup analysis (*N* = 476)**	• Patients from centres that delivered data on neonatal period<br>• Diagnosed in 2000-2019<br>• 0-19 years old when neonatal history was taken<br>• Definite PCD diagnosis

Table S2. Characteristics of PCD patients diagnosed after 2000 (N=1375).

**Characteristic**	***N* (%)**	**Age at Diagnosis (in years) Median (IQR)**	***N* (%) of Patients Diagnosed at 0–12m**
**Total**	1375 (100)	9.84 (3.92-16.26)	183 (13.3)
**Sex**			
Male	692 (50.3)	9.40 (2.87-15.72)	105 (15.2)
Female	683 (49.7)	10.35 (4.83-16.76)	77 (11.3)
**Country of residence**			
Argentina	101 (7.3)	6.00 (2.00-13.00)	14 (13.9)
Australia	43 (3.1)	8.30 (1.26-20.62)	10 (23.3)
Colombia	11 (0.8)	6.61 (4.04-8.34)	1 (9.1)
Cyprus	42 (3.1)	21.02 (10.25-43.25)	4 (9.5)
Czech Republic	51 (3.7)	9.71 (4.37-14.38)	6 (11.8)
Italy	303 (22.0)	9.27 (4.20-20.03)	50 (16.5)
Norway	35 (2.5)	1.42 (0.24-9.77)	16 (45.7)
Turkey	276 (20.1)	11.83 (8.26-15.61)	10 (3.6)
United Kingdom	513 (37.3)	9.40 (2.69-817.34)	71 (13.8)
**NRD**			
No	688 (50.0)	10.95 (5.96-16.85)	51 (7.4)
Yes	622 (45.2)	8.10 (1.62- 14.41)	128 (20.6)
No information	65 (4.7)	13.45 (4.07-34.20)	3 (4.6)
**Organ laterality**			
Situs solitus	761 (55.3)	11.84 (7.00-17.53)	44 (5.8)
Laterality defect	580 (42.2)	6.35 (1.11-13.63)	134 (17.6)
No information	34 (2.5)	8.82 (2.44-15.63)	4 (11.8)
**Clinical characteristics groups**			
No NRD, situs solitus	480 (34.9)	12.44 (7.64-17.96)	17 (3.5)
NRD, situs solitus	344 (25.0)	10.35 (4.88-17.14)	32 (9.3)
No NRD, laterality defect	252 (18.3)	8.77 (3.30-15.81)	36 (14.3)
NRD, laterality defect	305 (22.2)	4.46 (0.5-12.39)	97 (31.8)

NRD: neonatal respiratory distress.

Table S3. Age at diagnosis of PCD patients of all ages, per clinical characteristics group, based on reported NRD and organ laterality (N=1375).

**Clinical characteristics groups**	**Age at diagnosis N (%)**						
	**0–3m**	**3–12m**	**1–4y**	**5–9y**	**10–14y**	**> 14y**	**TOTAL**

**No NRD, situs solitus**	4 (0.8)	13 (2.7)	41 (8.6)	95 (19.9)	120 (25.2)	204 (42.8)	477 (100)
**NRD, situs solitus**	10 (2.9)	22 (6.4)	38 (11.1)	75 (21.9)	81 (23.7)	116 (33.9)	342 (100)
**No NRD, laterality defect**	8 (3.2)	28 (11.1)	32 (12.7)	61 (24.2)	50 (19.8)	73 (29.0)	252 (100)
**NRD, laterality defect**	43 (14.1)	54 (17.8)	51 (16.8)	52 (17.1)	46 (15.1)	58 (19.1)	304 (100)
**TOTAL**	65 (4.7)	117 (8.5)	162 (11.8)	283 (20.6)	297 (21.6)	451 (32.8)	1375 (100)

NRD: neonatal respiratory distress, m: months, yrs: years.

Age 0–2 months corresponds to 0–2.999 months, similar for all age groups.

Table S4. Prevalence of NRD and laterality defects in PCD patients aged 0-19 years with definite PCD diagnosis by country (N=476).

**Country**	**Neonatal presentation**			**Laterality defects**		
	**No NRD**	**NRD**	**Unknown**	**SS**	**LD**	**Unknown**
Argentina	8 (27%)	22 (73%)	0 (0%)	5 (17%)	25 (83%)	0 (0%)
Australia	6 (32%)	13 (68%)	0 (0%)	5 (26%)	14 (74%)	0 (0%)
Colombia	11 (100%)	0 (0%)	0 (0%)	10 (91%)	1 (9%)	0 (0%)
Cyprus	0 (0%)	5 (100%)	0 (0%)	1 (20%)	4 (80%)	0 (0%)
Czech Republic	14 (67%)	6 (29%)	1 (5%)	15 (71%)	6 (29%)	0 (0%)
Italy	54 (61%)	35 (39%)	0 (0%)	38 (43%)	51 (57%)	0 (0%)
Norway	4 (16%)	20 (80%)	1 (4%)	8 (32%)	17 (68%)	0 (0%)
Turkey	12 (39%)	19 (61%)	0 (0%)	19 (61%)	12 (39%)	0 (0%)
United Kingdom	84 (34%)	141 (58%)	20 (8%)	117 (48%)	110 (45%)	18 (7%)
**TOTAL**	193 (45%)	261 (5%)	22 (5%)	218 (46%)	240 (50%)	18 (4%)

NRD: neonatal respiratory distress, SS: situs solitus, LD: laterality defect.

Information presented as N (%).
